# Genomic molecular epidemiology of carbapenemase-producing *Escherichia coli* ST410 isolates by complete genome analysis

**DOI:** 10.1186/s13567-023-01205-6

**Published:** 2023-09-01

**Authors:** Su Min Kyung, Junho Lee, Eun-Seo Lee, Cheol-Yong Hwang, Han Sang Yoo

**Affiliations:** 1 Department of Infectious Diseases, College of Veterinary Medicine, Seoul National University, Seoul, Republic of Korea; 2 Department of Veterinary Dermatology, College of Veterinary Medicine, Seoul National University, Seoul, Republic of Korea

**Keywords:** Enterobacterales, metallo-β-lactamase, carbapenemase, NDM-5, *Escherichia coli*, ST410, NGS, MinION, epidemiology, One Health

## Abstract

The circulation of carbapenemase-producing *Escherichia coli* (CPEC) in our society is a serious concern for vulnerable patients in nosocomial environments. However, the genomic epidemiology of the circulation of CPEC bacteria among companion animals remains largely unknown. In this study, epidemiological analysis was conducted using complete genome identification of CPEC ST410 isolates obtained from companion animals. To estimate the genomic distance and relatedness of the isolates, a total of 37 whole-genome datasets of *E. coli* ST410 strains were downloaded and comparatively analysed. As a result of the analysis, the genomic structure of the chromosomes and plasmids was identified, revealing the genomic positions of multiple resistance and virulence genes. The isolates in this study were grouped into the subclade H24/RxC, with *fimH24*, and substituted quinolone resistance-determining regions (QRDRs) and multiple beta-lactamases, including extended-spectrum β-lactamase (ESBL) and carbapenemase. In addition, the in silico comparison of the whole-genome datasets revealed unidentified ST410 H24/Rx subgroups, including either high pathogenicity islands (HPIs) or H21 serotypes. Considering the genetic variations and resistance gene dissemination of the isolates carried by companion animals, future approaches for preventive measurement must include the “One Health” perspective for public health in our society.

**Supplementary Information:**

The online version contains supplementary material available at 10.1186/s13567-023-01205-6.

## Introduction

Nosocomial infections caused by carbapenemase-producing *Escherichia coli* (CPEC) are emerging as a major clinical threat. Dissemination of carbapenemase-producing bacteria among companion animals, which live close to humans in modern society, should be considered an urgent threat to public health [1, 2]. Carbapenem usage in animals is prohibited worldwide. However, the unauthorized usage of carbapenems is prevalent and not systematically monitored; thus, the dissemination of CPEC among companion animals is an unaddressed threat.

*E. coli* ST410, as an emerging opportunistic pathogen with both pathogenicity and resistance, is a successfully disseminating clone that spreads among humans, animals and the environment [3, 4]. It has been speculated that the *E. coli* ST410 strain first appeared in approximately the 1800s [5]. In the human clinical environment, carbapenemase-producing *E. coli* ST410 outbreaks have been reported in multiple countries, including Denmark [6, 7], Italy [8] and China [9]. In Germany, extended-spectrum β-lactamase (ESBL)-producing *E. coli* ST410 strains have been reported as circulating clones among humans, animals (wildlife and companion animals) and the environment [3, 10]. As emerging multidrug-resistant (MDR) pathogens, fluoroquinolone- and extended-spectrum cephalosporin (ESC)-resistant (i.e., B3/H24Rx) and carbapenem-resistant (i.e., B4/H24RxC) clades have been reported to be spreading in Europe and North America [5, 11].

In South Korea, a nationwide study investigated nosocomial strains isolated between 2011 and 2015 [12]. The investigation reported that *Klebsiella pneumoniae* carbapenemase-2 (KPC-2) and New Delhi metallo-β-lactamase-1 (NDM-1) were the dominant carbapenemase types in the Korean nosocomial environment. Thus far, ST410 strains have not been discovered in ongoing human investigations. To date, in South Korea, two separate studies have discovered a total of 7 strains of CPEC from companion animals [13, 14]. Unlike the results from the human investigation, all companion animal-derived CPEC strains harboured the IncX3 plasmid, which encodes *bla*_*NDM-5*_, and were identified as multilocus sequence type (MLST) ST410. The discrepancy between the findings in humans and animals could be attributed to insufficient data regarding companion animals or unidentified transmission events within our environment and animal community. However, given the growing importance of companion animals in human society, it is crucial to consider the possibility of human-animal transmission of CPEC pathogens.

MDR pathogenic bacteria, which have already become a major public health concern, are no longer confined to the realm of human health. As a natural phenomenon derived from ancient bacterial genomes [15], resistance genes can be shared and transferred through horizontal gene transfer among pathogenic bacteria in humans, animals, and the environment [16–18]. Therefore, it was necessary to conduct a resistance and virulence gene distribution analysis combining potential sources of shared yet undetected resistance genes while considering the comprehensive perspective of the “One Health” approach.

In this study, the genomic distance between companion animal-derived CPEC pathogens and previously identified strains was measured using whole-genome phylogenetic analysis. Since all the strains discovered in our country were identified solely as ST410, worldwide ST410 datasets were selected as the reference. To conduct the phylogenetic analysis, we performed whole-genome sequencing on four CPEC isolates obtained from companion dogs, including three isolates (DMCPEC2, DMCPEC3 and DMCPEC7) that were included in our previous study [13]. The whole-genome datasets were screened and analysed based on the public database to identify undiscovered genes that could be potential threats.

## Materials and methods

### Bacterial strain isolation and minimum inhibitory concentration

A total of 4 isolates of *E. coli* ST410 strains obtained from companion animals were included in this study. Three strains (DMCPEC2, DMCPEC3 and DMCPEC7), which were identified as carrying *bla*_NDM-5_-encoding IncX3 plasmids in a previous study [13], were included among the four isolates. NB7CPEC was isolated from a screening rectal swab at a local veterinary clinic in Seoul, South Korea. Meropenem-impregnated (1 μg/mL) MacConkey (MIM) agar was used to identify the carbapenem-resistant gram-negative phenotype from the rectal swab of a mixed Pomeranian dog. The host dog (6-year-old mixed Pomeranian canine, spayed female) had no specific clinical disease condition and was swabbed for carbapenemase screening with a normal rectal swab collected by professional veterinarians in accordance with the Guide for the Care and Use of Laboratory Animals and the Animal Welfare Act. The antimicrobial resistance minimum inhibitory concentration (MIC) profile was determined using the broth microdilution method. *E. coli* strain ATCC 25922 was used as a quality control strain for the MIC determination, following the Clinical and Laboratory Standards Institute (CLSI) recommendations for performance and interpretation [19].

### Total DNA isolation followed by in vitro genotyping

The Wizard Genomic DNA purification kit (Promega, Madison, WI) was used for total DNA purification, and carbapenemase gene screening was performed using previously designed multiplex PCR primers and protocols [20]. To compare the sequences with available GenBank data, Sanger sequencing was performed using the Basic Local Alignment Search Tool (BLAST) network service [21].

Bacterial species were determined using a matrix-assisted laser desorption ionization–time of flight-mass spectrometry (MALDI–TOF–MS; Bruker Daltonik GmbH, Bremen, Germany) biotyper and 16S rRNA sequencing.

Classical MLST was performed using a previously described protocol [22] to evaluate seven housekeeping genes (*adk*, *fumC*, *gyrB*, *icd, mdh*, *purA* and *recA*), and the results were further confirmed on the online database [23].

### Combined complete genome sequencing and de novo assembly

The whole-genome DNA samples of 4 isolates of CPEC ST410 were purified with a Wizard Genomic DNA purification kit (Promega, Madison, WI, USA) from overnight cultures. For high-quality sequencing and assembly, both long and short genomic DNA libraries were prepared. Short-read sequencing was performed using an Illumina NovaSeq 6000 (Illumina, San Diego, CA, USA) platform following a paired-end 2 × 150-bp protocol. The Oxford Nanopore platform (Oxford Nanopore Technologies, Oxford, UK) was employed for long-read sequencing. The ONT library was constructed and sequenced by using the Ligation Sequencing Kit (SQK-LSK109), the Flow Cell Priming Kit (EXP-FLP002) and Flowcell (FLO-MIN106).

Basecalling and demultiplexing of barcodes were conducted with Guppy basecaller and barcoder v6.0.7 [24], followed by combined trimming and filtering of the reads with FiltLong v0.2.0 [25]. De novo assembly was performed with Flye v2.8.3 [26], and the results were combined with short reads using the Unicycler v0.4.8 hybrid assembler [27]. Examinations for overlaps and circularization were performed with Circlator v1.5.5 [28]. Pilon v1.23 [29] was employed for data polishing. The genome completeness for the generated assembled sequences was confirmed through calculations using BUSCO v4.1.2 [30]. Genome structural annotation was conducted using Prokka v1.14.6 [31], and functional annotation was performed with DIAMOND v 0.9.30 [32] and Blast2GO v4.1.9 [33] to perform gene ontology [34] analysis.

### In silico typing and identification for bioinformatic comparison

For comparative analysis, 37 genomic datasets of *E. coli* ST410 strains were downloaded from the National Center for Biotechnology Information [35] and compared with the 4 isolates sequenced in this study. The assembled genomes were screened for comparison of the resistance genes, virulence genes, serotypes, SPI sites, MLST, plasmid types and *fimH* types on the Center for Genomic Epidemiology (CGE) server [36] for in silico utilization of ResFinder 4.1, VirulenceFinder 2.0, SerotypeFinder 2.0, SPIFinder 2.0, MLST 2.0, PlasmidFinder 2.1 and FimTyper 1.0. The schematic complete genome structure maps of the chromosomes and plasmids were generated with the CGView server [37]. High-quality whole-chromosome SNPs were identified from the chromosomal reference sequence of YD786 (GenBank no. CP013112.1) for concatenated alignment using the standard settings of CSI Phylogeny [38]. A maximum likelihood (ML) tree was constructed with 1000 bootstrap replicates in MEGA 11 software [39]. The comparative gene distribution annotations and heatmaps were annotated, and a phylogenetic tree was constructed and visualized on iTOLs [40].

## Results

### Isolate profiles and minimum inhibitory concentrations

The NDM-5-harbouring *E. coli* ST410 isolate NB7CPEC was discovered from a rectal swab of a 6-year-old mixed Pomeranian canine (Table 1) hospitalized in a local veterinary clinic. The minimum inhibitory concentration value was measured for NB7CPEC and combined with the MIC results from a previous study (DMCPEC2, DMCPEC3 and DMCPEC7). All 4 isolates were identified as resistant strains against 3 tested carbapenems (Additional file 1), namely, ertapenem (MIC value ≥ 32 μg/mL, ≥ 2 μg/mL as the resistance breakpoint standard), imipenem (MIC value ≥ 16 μg/mL, ≥ 4 μg/mL as the resistance breakpoint standard) and meropenem (MIC value ≥ 32 μg/mL, ≥ 2 μg/mL as the resistance breakpoint standard) [19]. The isolates also showed high MIC values against ceftazidime, cefepime (3rd- and 4th-generation cephalosporins, respectively), gentamicin (aminoglycosides), ampicillin (aminopenicillins) and tetracycline (tetracyclines). The MIC of tobramycin (aminoglycosides), chloramphenicol (phenicols), and trimethoprim (folate pathway inhibitors) showed different values according to the isolates. The only susceptible option in common was colistin (MIC values lower than 0.5 μg/mL) for the CPEC isolates.

**Table 1:** **Basic information of** ***bla***_**NDM-5**_**-encoded CPEC isolates included in this study**

Bacterial strain	Bacterial species	Carbapenemase type	MLST	Host	Year of isolation	Isolation source
DMCPEC2	*Escherichia coli*	NDM-5	410	Canine mixed	2019	Urine
DMCPEC3	*Escherichia coli*	NDM-5	410	Canine Labrador Retriever	2020	Ear swab
DMCPEC7	*Escherichia coli*	NDM-5	410	Canine Bichon Frise	2019	Ear swab
NB7CPEC	*Escherichia coli*	NDM-5	410	Canine mixed Pomeranian	2021	Rectal swab

The basic profiles of bacterial strains carrying *bla*_NDM-5_-encoded IncX3 plasmids were described. Four isolates were discovered from a veterinary clinical hospital. The basic bacterial genotypes and host information are listed.

### Qualification of the sequenced whole-genome datasets

The 4 *E. coli* ST410 isolates identified from 4 different companion dogs were subjected to whole-genome sequencing, and a high-quality nucleotide sequence was generated (Additional file 2). Whole-genome sequencing identified chromosomes and 2 plasmids from each strain. In NB7CPEC, 2 additional plasmids were identified (Table 2).

**Table 2:** **Assembled and identified genomes in this study**

Bacterial strain	Gene type	Gene length (bp)	Gene form	GC contents	CDS	rRNA	tRNA
DMCPEC2	Chromosome	4 832 084	Circular	50.66	4534	22	87
	Plasmid (FII_FIA_FIB)	77 205	Circular	51.27	82	0	0
	Plasmid (X3)	46 159	Circular	46.66	60	0	0
DMCPEC3	Chromosome	4 736 397	Circular	50.59	4384	22	87
	Plasmid (FII_FIA_FIB)	82 232	Circular	51.47	87	0	0
	Plasmid (X3)	46 749	Circular	46.64	58	0	0
DMCPEC7	Chromosome	4 715 539	Circular	50.64	4374	22	87
	Plasmid (FII_FIA_FIB)	77 205	Circular	51.27	82	0	0
	Plasmid (X3)	46 159	Circular	46.65	60	0	0
NB7CPEC	Chromosome	4 759 653	Circular	50.62	4419	22	87
	Plasmid (p0111)	96 439	Circular	47.86	111	0	3
	Plasmid (FII_FIA_FIB)	80 707	Circular	50.81	87	0	0
	Plasmid (FII(pHN7A8))	73 618	Circular	52.12	87	0	1
	Plasmid (X3)	46 160	Circular	46.65	59	0	0

Each strain contained a single chromosome. Two types of plasmids were carried by all isolates in common, including NDM-5 harbouring the IncX3 plasmid. NB7CPEC was identified with 2 additional plasmids, which were typed as IncFII (pHN7A8) and p0111.

### Identification of characteristic genes in the chromosomes and plasmids of the ST410 strains

**Figure 1:**
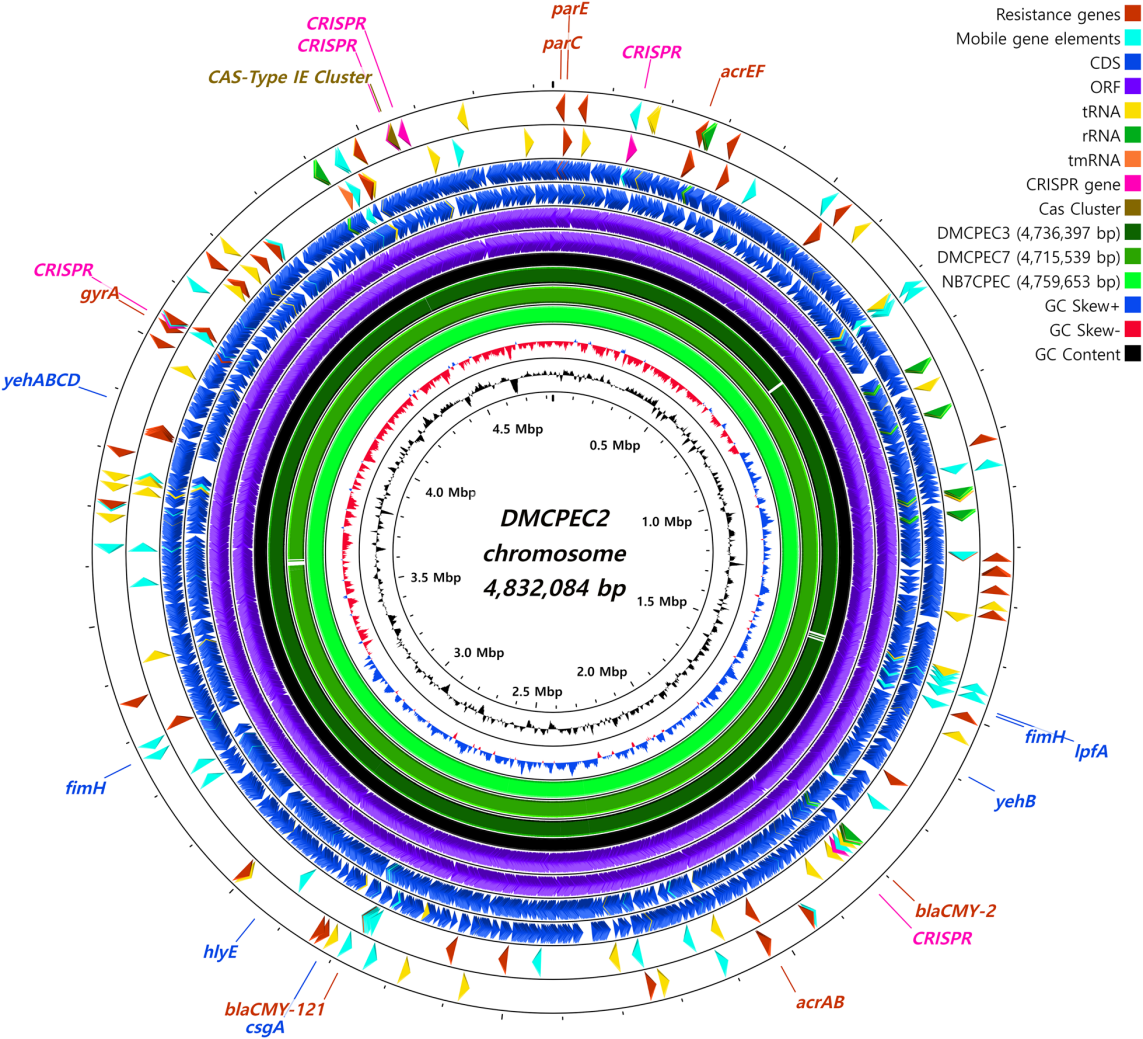
**Circular map of comparative chromosomes of the 4 ST410 isolates identified in this study.** The whole chromosomes of the *E. coli* ST410 strains identified in this study were comparatively mapped. The chromosomal map of DMCPEC2 (4 832 084 bp) was utilized as the backbone for visualization and is depicted as a black circle. The circular map was generated by using CGView.

The identified chromosomal length of each strain was between 4.71 and 4.83 Mbp (Figure 1). The ESBL-encoding genes of CMY-2 and CMY-121 were identified. The chromosomes included variant sites of quinolone resistance-determining regions (QRDRs) of the *gyrA* (S83L and D87N), *parC* (S80I) and *parE* (S458A) genes. A total of 5 CRISPR region sites were also discovered, with a CAS-type IE cluster in the chromosome. The virulence genes (*csgA*, *fimH*, *lpfA*, *hlyE* and *yehABCD*) were also identified, and their positions were marked on the visualization map.

**Figure 2:**
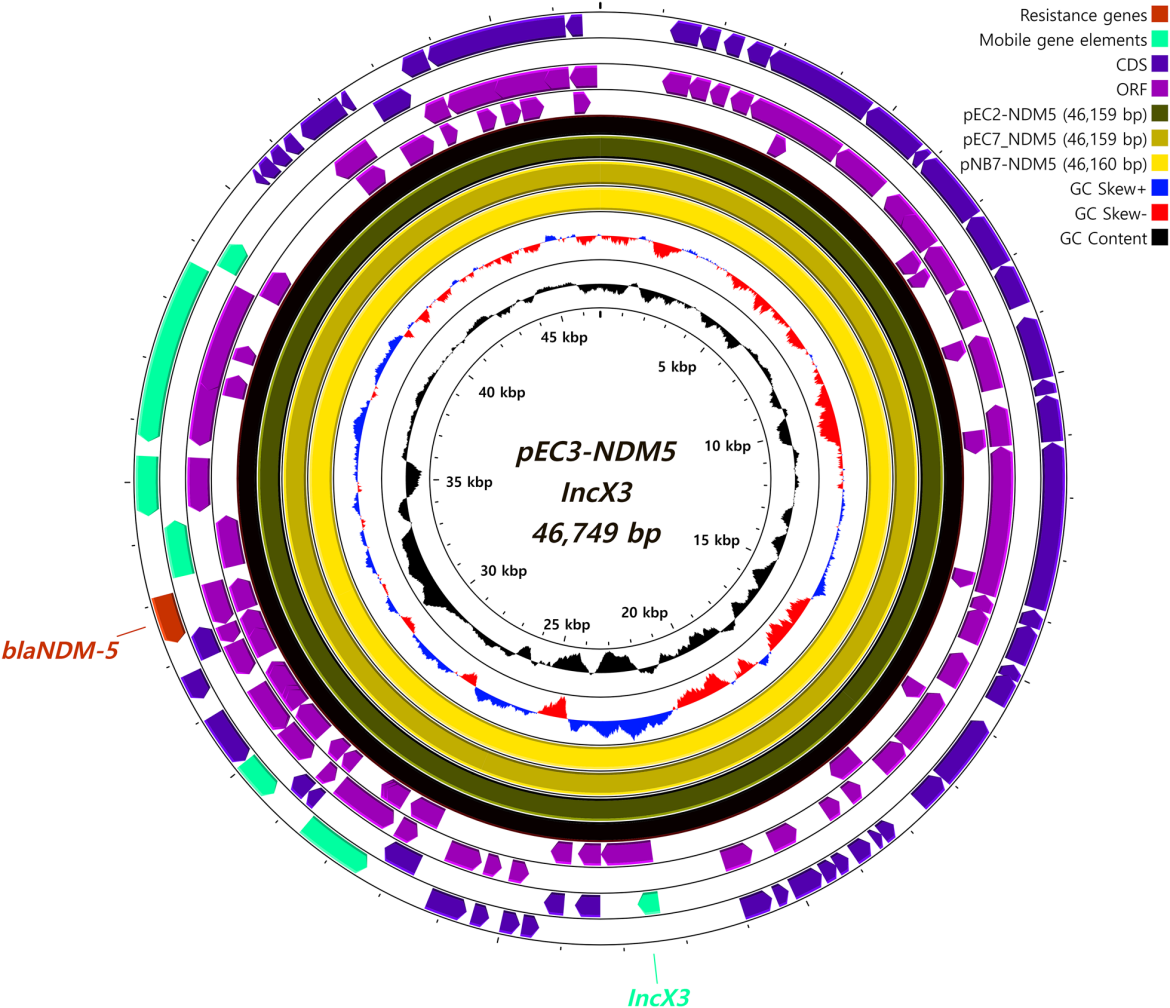
**Schematic visualization map of IncX3 plasmids encoding *bla***_***NDM-5***_** carried by ST410 strains.** The approximately 46 kbp-long IncX3-type plasmids encoding the carbapenemase gene *bla*_*NDM-5*_ were carried by all 4 strains. The IncX3 plasmids were consistent with the datasets reported in a previous study [13]. The data for pNB7-NDM5 were included in the visual map. The plasmid map of pEC3-NDM5 (46 749 bp) was used as the backbone for visualization and is depicted as a black circle. The circular map was generated by using CGView.

**Figure 3:**
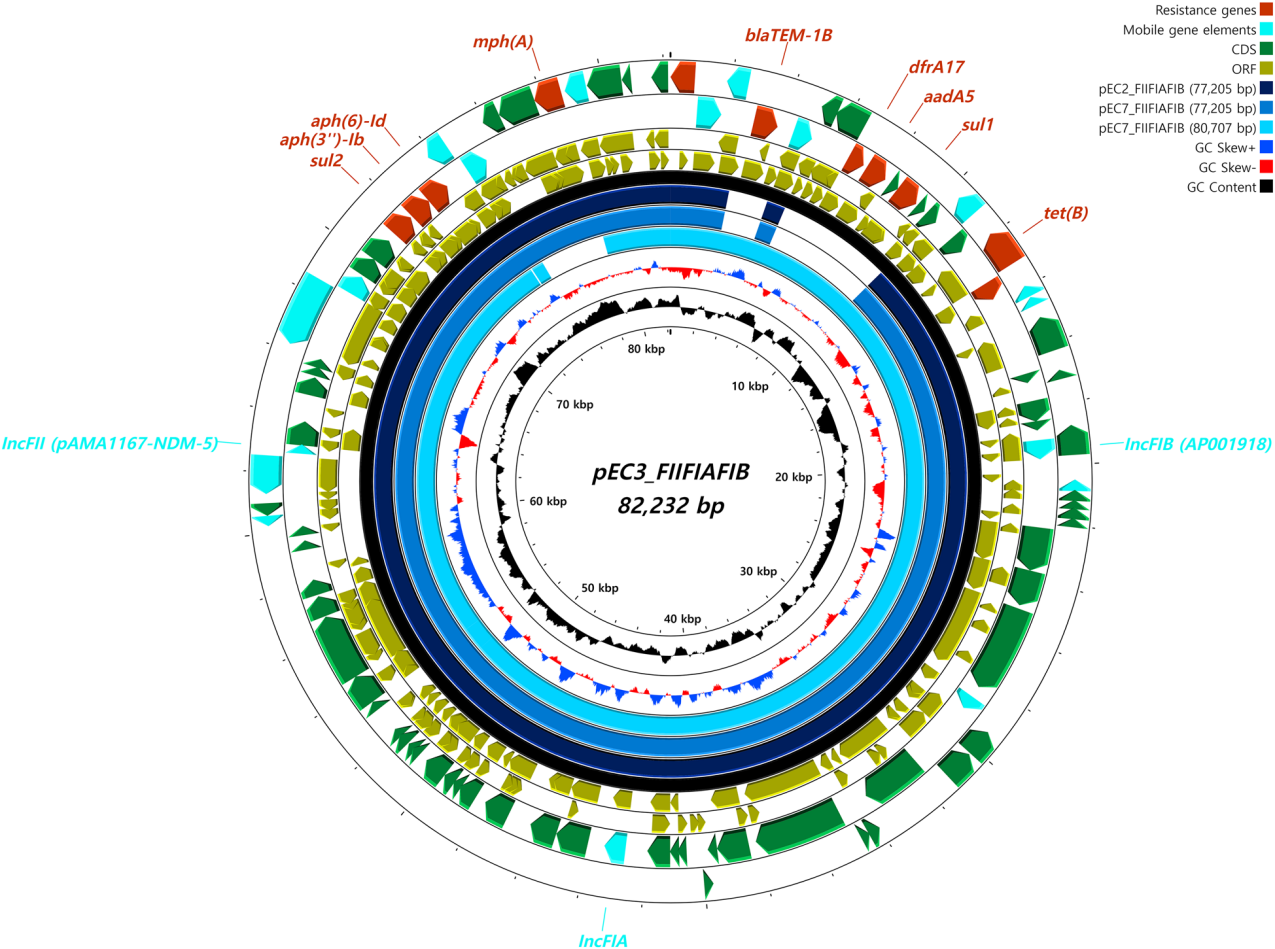
**Integrated map of the 3 plasmid types carried by the ST410 strains.** Three distinct plasmid types (namely, FII (pAMA1167-NDM-5), FIA and FIB (AP001918)) were integrated into 77–82 kbp-long plasmids. The plasmid map of pEC3-FIIFIAFIB (82 232 bp) was used as the backbone for visualization and is depicted as a black circle. The circular map was generated by using CGView.

Two heterogeneous plasmids were carried by all isolates, including the *bla*_*NDM-5*_-encoding IncX3 plasmid (Figure 2). The other plasmid (Figure 3) was discovered as an integrated form of three types of plasmids, namely, IncFIA (% identity; 99.74), IncFIB (AP001918) (% identity; 98.39) and IncFII (pAMA1167-NDM-5) (% identity; 100).

**Figure 4:**
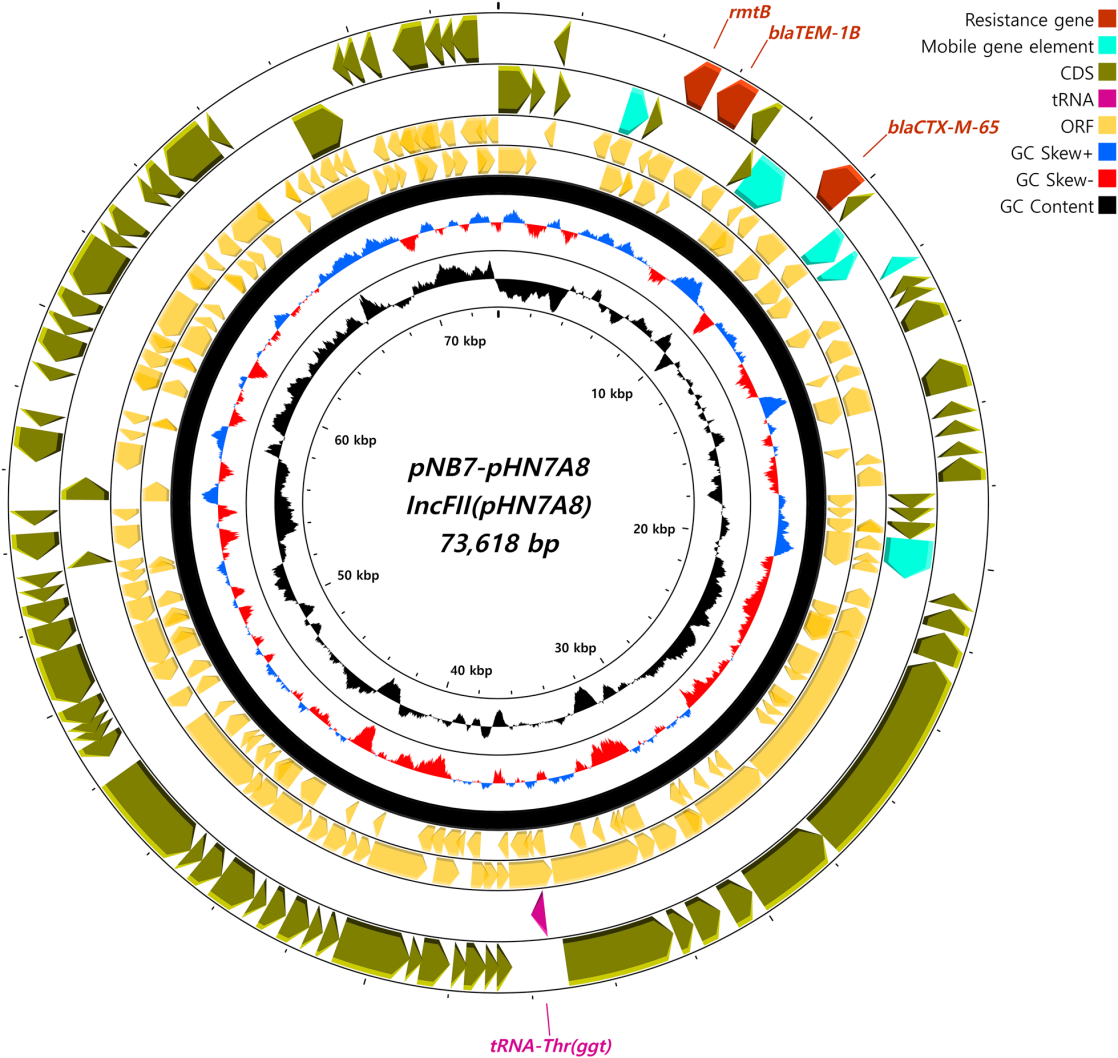
**Circular map of the IncFII (pHN7A8)-type plasmid carried by strain NB7CPEC.** The plasmid pNB7-pHN7A8 was identified from NB7CPEC and encoded the ESBL genes *bla*_*TEM-1B*_ and *bla*_*CTX-M-65*_. The circular map was generated by using CGView.

**Figure 5:**
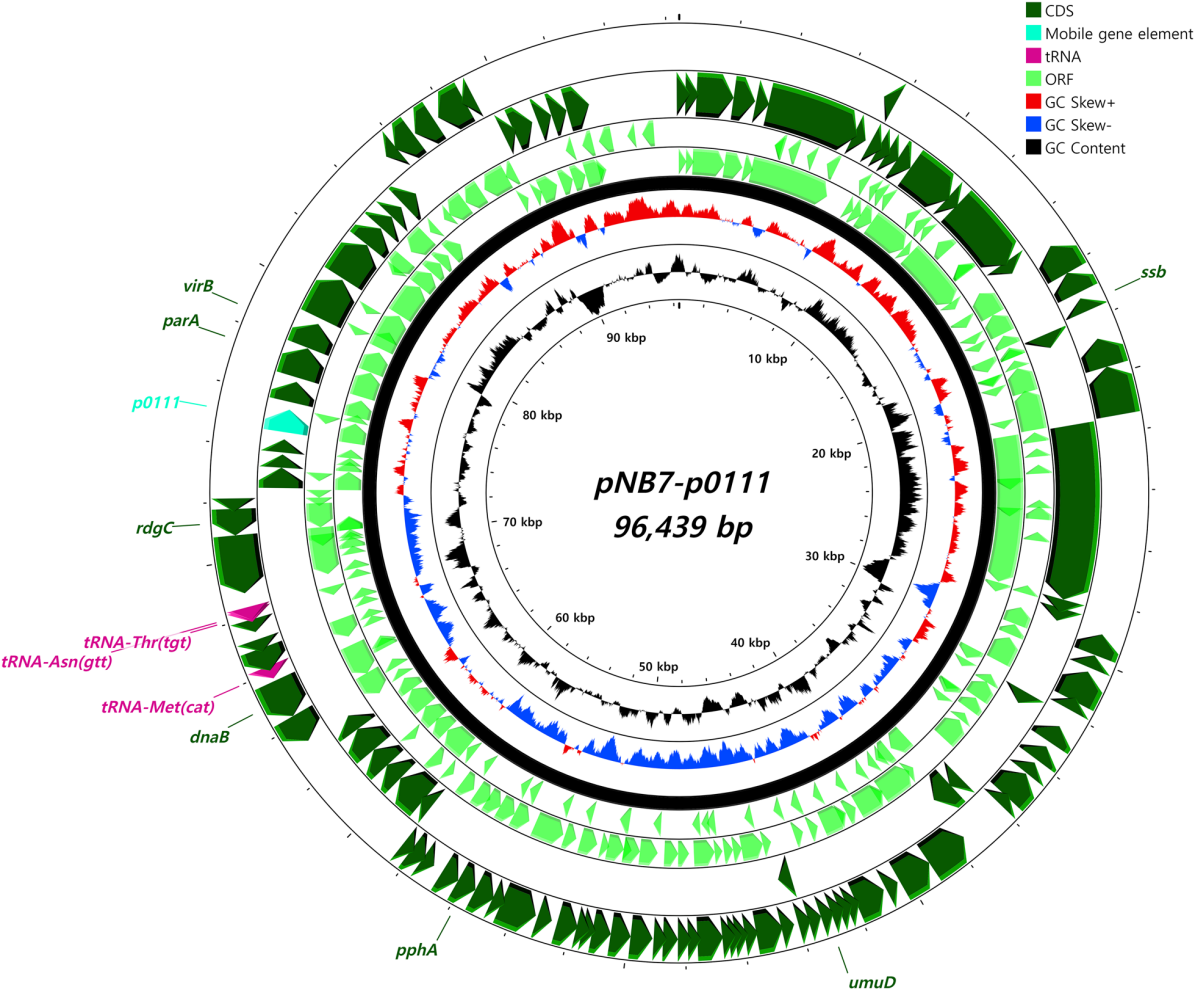
**Map of the p0111-type plasmid carried by the ST410 strain NB7CPEC.** The p0111-type plasmid was identified without additional resistance genes. The circular map was generated by using CGView.

The bacterial strain NB7CPEC, which was isolated in 2021 from a local veterinary clinic, carried an additional 2 different plasmids (Figures 4 and 5). The IncFII (pHN7A8)-type plasmid (Figure 4) was identified as a 73 618 bp-long plasmid encoding the ESBL-harbouring genes *bla*_*TEM-1B*_ and *bla*_*CTX-M-65*_. The structural positions of the resistance genes and the mobile gene elements were mapped on the schematic map of the whole plasmid. The p0111-type (% identity, 98.08) plasmid was also identified as a 96,439 bp-long plasmid (Figure 5), although it lacked mobile gene cassettes and resistance genes.

### Whole-genome phylogeny and bioinformatic comparison of the characteristic genes

The complete genome datasets of 37 *E. coli* ST410 strains isolated between 2010 and 2020 from 17 different countries were downloaded and included in a complete genome comparison along with the data from this study (Additional file 3). The strains were isolated from China (*n* = 8), South Korea (*n* = 5, including 4 isolates from this study), the USA (*n* = 4), Switzerland (*n* = 4), the United Kingdom (*n* = 3), Brazil (*n* = 2), Germany (*n* = 2), Ghana (*n* = 2), India (*n* = 2), Malaysia (*n* = 2), and Canada, Cuba, Denmark, France, Norway, Portugal and Spain (*n* = 1 each).

The measured number of valid SNP positions of the whole chromosomes identified by the CSI Phylogeny pipeline was 5271. The pairwise SNP difference, adjusted according to YD786 (GenBank no. CP013112.1) as the reference genome, ranged from 0 (between CP024801 and CP026473) to 2,204 (between CP031231 and CP027205) (Additional file 4). Among the isolates from this study, the pairwise SNP differences were from 28 (between DMCPEC3 and DMCPEC7) to 533 (between DMCPEC2 and NB7CPEC). The input parameters, analysis quality and identity of each strain against the reference are displayed in Additional file 5. The resistance and virulence genes and plasmid types are denoted in Additional files 6, 7, 8.

**Figure 6:**
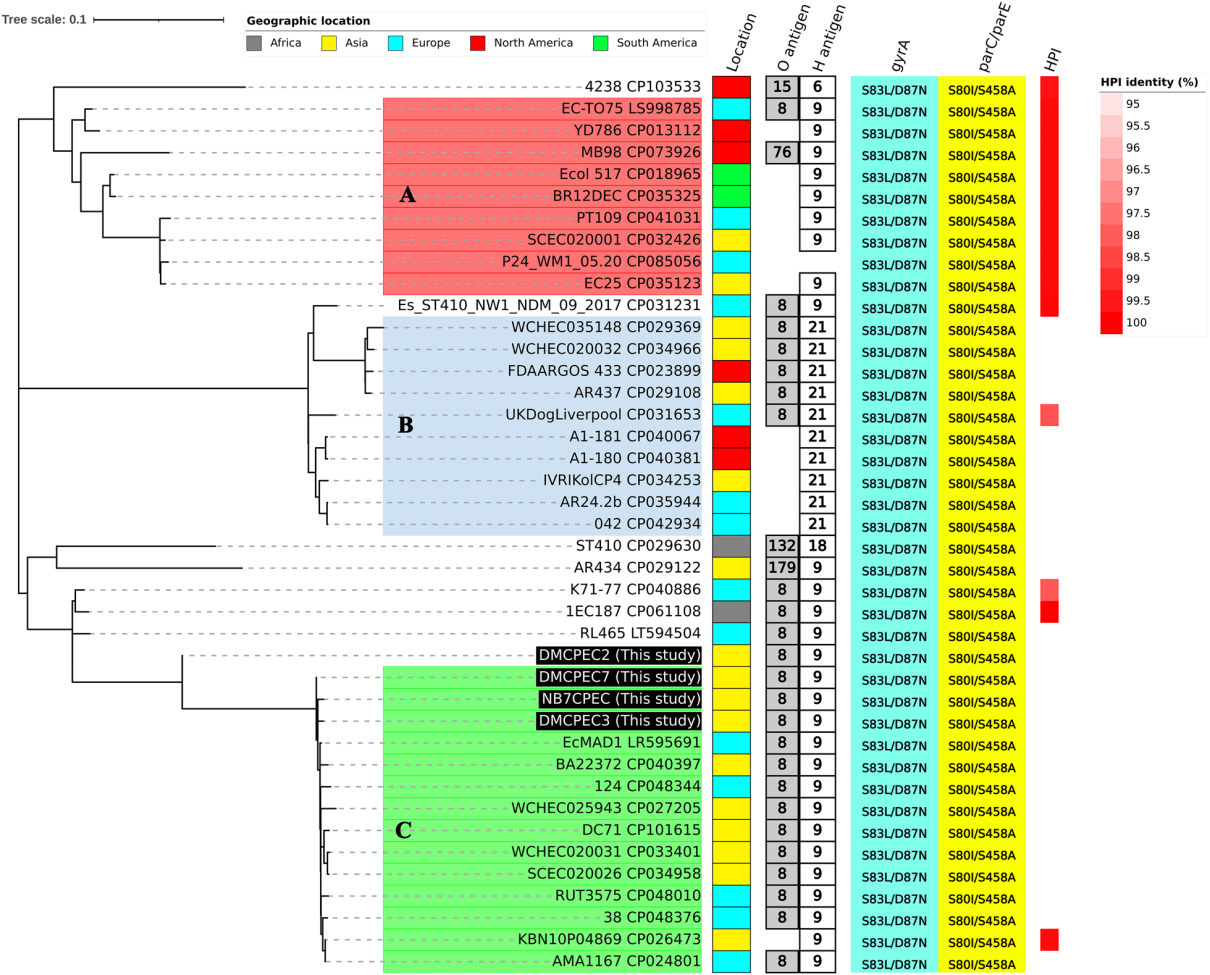
**Epidemiological comparison of the complete genome datasets of 41 ST410 strains.** A maximum likelihood (ML) tree was constructed and visualized by iTOLs based on whole-chromosome SNPs. The isolates originating from this study are highlighted with a black background. The coloured ranges covering the strain labels show the identified subgroups: Group A (red); Group B (blue); Group C (green). The coloured and labelled columns indicate the epidemiological information of the ST410 strains. From left to right, geographic locations; O antigen types; H antigen types; quinolone resistance-determining regions (QRDRs) variation sites of *gyrA*; QRDR variation sites of *parC/parE*; levels of identity of *salmonella* pathogenicity islands (%). The geographic locations of the strains are denoted in distinguishable colours according to their continents of origin: Africa (grey), Asia (yellow), Europe (cyan), North America (red) and South America (green).

A whole-chromosome SNP-based phylogenetic tree was then constructed and displayed along with the epidemiological datasets (Figure 6). All 41 isolates included amino acid substitutions in the QRDR and *fimH24* genes, which could be classified as the B/H24R lineage.

The analysis of the subclade ST410 B/H24R identified the following distinguishable groups: (i) Group A, including the H9 antigen and the type 1 high-pathogenicity island (HPI) of the *Salmonella* pathogenicity islands, (ii) Group B, identified with the H21 antigen, and (iii) Group C (B4/H24RxC), carrying the ESBLs and carbapenemase genes. Three of the strains from this study (DMCPEC3, DMCPEC7 and NB7CPEC) encoding *bla*_*CMY-6*_ and *bla*_*NDM-5*_ could be included in Group C.

The isolates included in phylogenetic group A included HPIs. The discovered HPI gene dataset was identical to the type 1 HPI (identity > 98%) identified from *Salmonella enterica* group VI [41]. The SNP differences ranged from 37 (between CP018965 and CP035325) to 662 (between CP035123 and CP073926). Several virulence genes were identified from Group A with high priority, namely, *iucC* (aerobactin synthetase), *ituA* (ferric aerobactin receptor) and *sitA* (iron transport protein). The tetracycline resistance gene *tet(A)* was carried by the isolates of Group A with higher priority than the other groups.

Group B featured the H21 antigen type. The isolates of Group B carried the *bla*_*CMY-42*_ gene with relatively higher priority than the other groups. The differences in SNPs ranged from 5 (between CP035944 and CP042934) to 403 (between CP031653 and CP029369).

**Figure 7:**
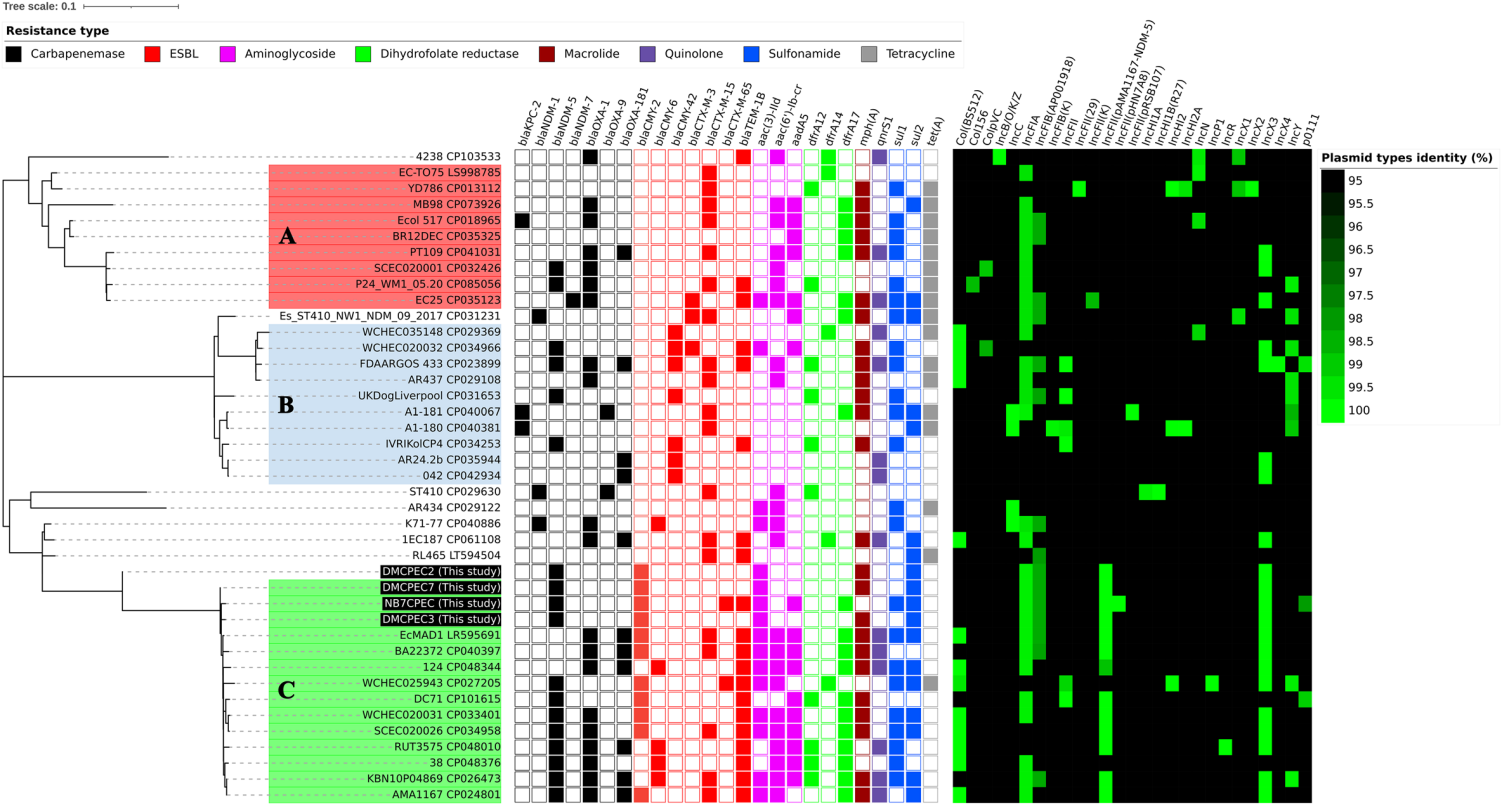
**Whole-genome dataset comparison of the antimicrobial resistance genes and plasmid types identified from the ST410 strains.** Different types of antimicrobial resistance genes were categorized and are shown in distinguishable colours and rectangular boxes: carbapenemases (black), ESBLs (red), aminoglycosides (magenta), dihydrofolate reductases (green), macrolides (brown), quinolones (purple), sulfonamides (blue) and tetracyclines (grey). A heatmap indicating the identified plasmid types is shown along with the antimicrobial resistance gene distribution map.

The isolates in Group C mainly originated from samples from Asia and Europe. The serotypes of the isolates in Group C were identified as O8:H9. The pairwise SNP difference matrix ranged between 15 (CP034958 and CP033401) and 103 (CP048344 and CP027205). Group C isolates were found to have carbapenemase genes (Figure 7), encoding at least one carbapenemase gene of NDM-5 or OXA-1. The ESBL genes of CMY-2, CMY-6 and TEM-1B were discovered in this group with higher priority, along with the IncFIA-, IncFII- and IncX3-type plasmids.

## Discussion

**Figure 8:**
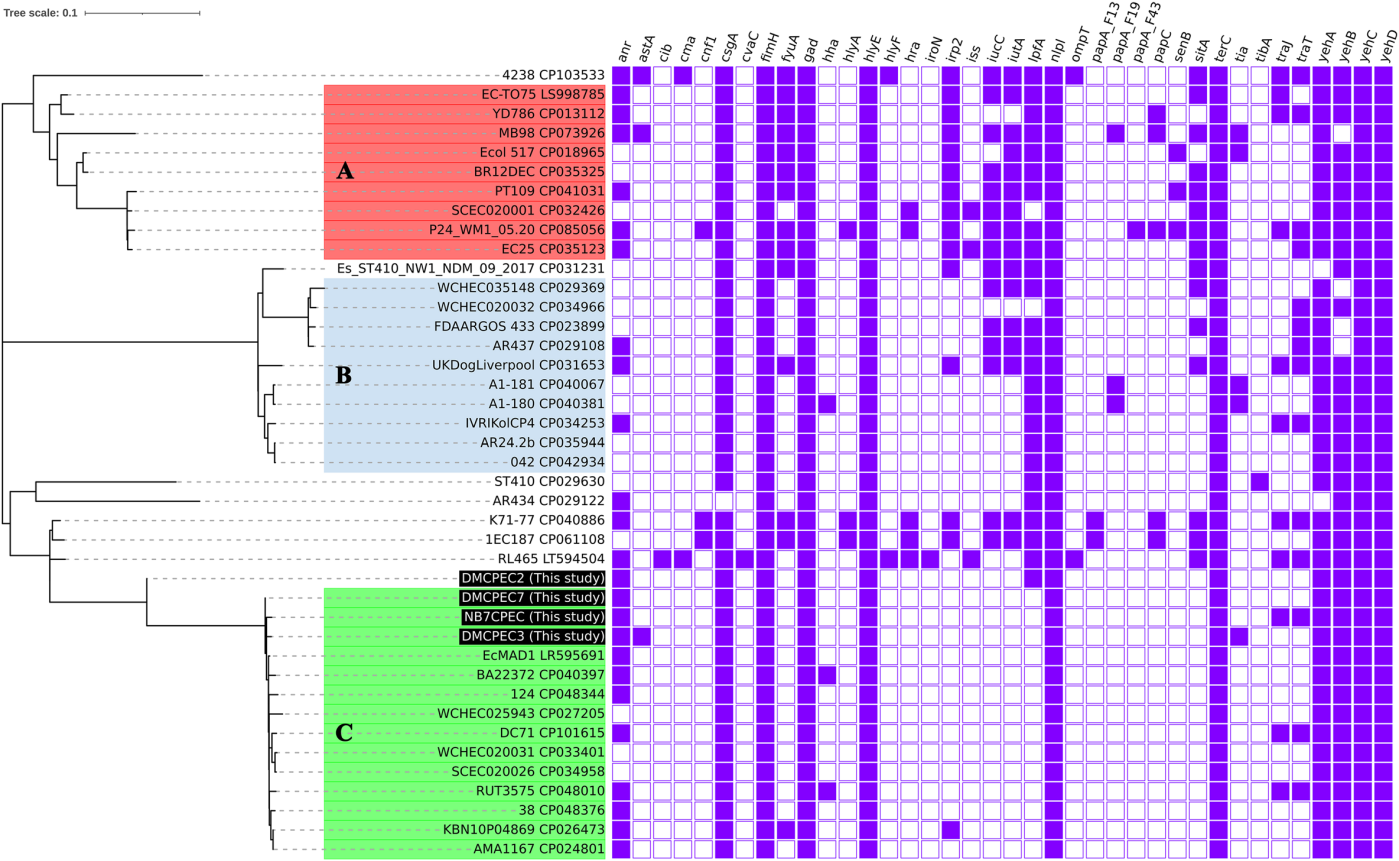
**Virulence gene distribution comparison identified from the whole genome datasets.** Virulence genes were screened from the whole genome datasets adjusting VirulenceFinder 2.0 via the CGE server and visualized using iTOLs. Distinguished phylogenetic subgroups are indicated with coloured ranges: Group A (red); Group B (blue); Group C (green).

The extraintestinal pathogenic *E. coli* ExPEC ST410 strain is known to circulate not only in humans but also in animals (wildlife and companion animals) and the environment [3, 10]. Through whole-genome phylogenetic analysis, it was discovered that the companion animal-derived isolates were closely grouped with strains identified from humans, animals and the environment (in subgroup C, Figures 6–8). The total number of whole-genome SNP differences of isolate DMCPEC7, carried by a companion dog, from the human blood isolate KBN10P04869 (GenBank no. CP026473) was 36. Previous investigations on Korean isolates have indicated differences in genetic characteristics, such as MLST types, between human and animal strains. The MLST types of the human-obtained CPEC isolates investigated by the national laboratory surveillance system were ST131, ST1642 and ST101, whereas ST410 was not reported [12]. In contrast, all CPEC isolates identified from companion animals in South Korea thus far have been typed as ST410. Despite the phylogenetic relatedness, the genetic studies conducted in this work are not suitable to provide direct evidence of the transmission between the isolative sources of those strains, including human-animal transmission. However, when considering the genetic evidence revealed by the whole-genome phylogeny, it is crucial to seriously consider the possibility of circulation among humans, animals and the environment. Therefore, an integrative “One Health” approach should be applied for control measures considering the *E. coli* ST410 strains.

The *E. coli* ST410 strains, along with ST131, have been proposed as globally circulating strains of extraintestinal pathogenic *E. coli* (ExPEC) [10]. ExPEC bacteria are known to encode various extraintestinal virulence factors and have been attributed to various infectious diseases, including neonatal meningitis, urinary tract infections, bloodstream infections and pneumonia [42–44]. In these clinical situations, the administration of β-lactam antibiotics, including carbapenems, is an important treatment option. However, ExPEC bacteria may be resistant not only to β-lactam antibiotic agents due to the production of various β-lactamases, such as ESBL and carbapenemase but also to other antibiotics, including fluoroquinolones, tetracyclines and aminoglycosides [45–47]. In this study, companion animal-derived ST410 strains were found to carry various virulence genes and antimicrobial resistance genes. The MIC results (Additional file 1) revealed that colistin is currently the only viable option for treating these isolates. Therefore, the circulation of these strains in companion animals within our community should be addressed seriously, considering that these strains have the capacity to not only cause extraintestinal infections as ExPEC bacteria but also resist various antimicrobial agents as CPEC pathogens.

*E. coli* strains are largely classified into phylogroups A, B1, B2, C, D, E or F, followed by further categorization of sequence types, clades and subclades [48]. The carbapenemase gene is mainly carried by *E. coli* strains in phylogroups A and B1 [49], and *E. coli* ST410 strains are grouped into phylogroup A. ST410 has been reported as a high-risk clone in previous studies [48]. The subclade of ST410 carrying *fimH24* has been classified by B2/H24R (*gyrA* and *parC* mutations), B3/H24Rx (additional carriage of the ESBL gene) and B4/H24RxC (carbapenemase introduction) [5]. In this study, further specific groups were identified in the B2/H24R subclade by bioinformatic gene identification based on whole-genome data. In particular, the distribution of virulence factor genes (Figure 8) was greater in Group A strains relative to the other strains, such as HPIs, *iucC*, *ituA* and *sitA*. HPI and ExPEC bacteria are strongly correlated and have been described as the causative agents of various extraintestinal infections [50, 51]. Conversely, Group C isolates were identified as having a relatively larger dissemination of antimicrobial resistance genes. To continue to define the genetic characteristics of the ST410 strains identified in this study, further investigations are needed, such as additional isolate collection and antimicrobial and virulence phenotyping.

Whole-genome analyses revealed the genomic potential of CPEC isolates identified from companion animals, indicating that these strains should be considered a potential threat to public health. Therefore, it is crucial to consider new measures for controlling the dissemination of CPEC ST410 strains in our society, adopting a combined public health approach with a “One Health” perspective.

### Supplementary Information

**Additional file 1: MIC profiles of the ST410 strains**.

**Additional file 2: Whole-genome profiles of sequenced** ***E. coli*** **strains**.

**Additional file 3: Epidemiological profiles of analysed whole-genome datasets in this study**.

**Additional file 4: The pairwise SNP difference matrix of whole-chromosome datasets extracted from the reference genome**.

**Additional file 5: Quality of SNP extraction analysis, including input parameters and identity of each strain with the reference**.

**Additional file 6: The dissemination metadata of antimicrobial resistance gene carriage in the whole-genome datasets, searched by in silico screening by the ResFinder 4.1 database**.

**Additional file 7: Genomic metadata of virulence genes (presence or absence), serotypes, Salmonella pathogenicity islands (SPIs) and FimH types searched on the Center for Genomic Epidemiology (CGE) server**.

**Additional file 8: Plasmid-type metadata identified by PlasmidFinder 2.1 and presented as gene identity (%)**.

## Data Availability

All referred sequences in this study are available from the NCBI BioProject number PRJNA858561. All data generated or analysed during this study have been submitted with this manuscript. All genetic information of the plasmids was deposited in GenBank. Therefore, all data from this study are available to the public.

## Abbreviations

BLAST: Basic Local Alignment Search Tool
CPEC: carbapenemase-producing *Escherichia coli*
CGE: Center for Genomic Epidemiology
CLSI: Clinical and Laboratory Standards Institute
CRE: carbapenem-resistant Enterobacterales
ESBL: extended-spectrum β-lactamase
ESC: extended-spectrum cephalosporin
ExPEC: extraintestinal pathogenic *E. coli*
GO: Gene Ontology
Inc: incompatibility type
MALDI-TOF: matrix-assisted laser desorption ionization–time of flight-mass spectrometry
MDR: multidrug resistant
MIC: minimum inhibitory concentration
MIM: meropenem-impregnated MacConkey
ML: maximum likelihood
MLST: multilocus sequence type
ORF: open reading frame
QRDR: quinolone resistance-determining region
SNP: single nucleotide polymorphism
SPIs: *Salmonella* Pathogenicity islands

## References

[1] Shaheen BW, Nayak R, Boothe DM (2013). Emergence of a New Delhi metallo-β-lactamase (NDM-1)-encoding gene in clinical *Escherichia coli* isolates recovered from companion animals in the United States. Antimicrob Agents Chemother.

[2] Stolle I, Prenger-Berninghoff E, Stamm I, Scheufen S, Hassdenteufel E, Guenther S, Bethe A, Pfeifer Y, Ewers C (2013). Emergence of OXA-48 carbapenemase-producing *Escherichia coli* and *Klebsiella pneumoniae* in dogs. J Antimicrob Chemother.

[3] Falgenhauer L, Imirzalioglu C, Ghosh H, Gwozdzinski K, Schmiedel J, Gentil K, Bauerfeind R, Kämpfer P, Seifert H, Michael GB (2016). Circulation of clonal populations of fluoroquinolone-resistant CTX-M-15-producing *Escherichia coli* ST410 in humans and animals in Germany. Int J Antimicrob Agents.

[4] Guenther S, Ewers C, Wieler LH (2011). Extended-spectrum beta-lactamases producing *E. coli* in wildlife, yet another form of environmental pollution?. Front Microbiol.

[5] Roer L, Overballe-Petersen S, Hansen F, Schønning K, Wang M, Røder BL, Hansen DS, Justesen US, Andersen LP, Fulgsang-Damgaard D (2018). *Escherichia coli* sequence type 410 is causing new international high-risk clones. Msphere.

[6] Roer L, Hansen F, Thomsen MCF, Knudsen JD, Hansen DS, Wang M, Samulioniené J, Justesen US, Røder BL, Schumacher H (2017). WGS-based surveillance of third-generation cephalosporin-resistant *Escherichia coli* from bloodstream infections in Denmark. J Antimicrob Chemother.

[7] Overballe-Petersen S, Roer L, Ng K, Hansen F, Justesen US, Andersen LP, Stegger M, Hammerum AM, Hasman H (2018). Complete nucleotide sequence of an *Escherichia coli* sequence type 410 strain carrying *bla*_*NDM-5*_ on an IncF multidrug resistance plasmid and bla OXA-181 on an IncX3 plasmid. Genome Announc.

[8] Piazza A, Comandatore F, Romeri F, Pagani C, Floriano AM, Ridolfo A, Antona C, Brilli M, Mattioni Marchetti V, Bandi C (2018). First report of an ST410 OXA-181 and CTX-M-15 coproducing *Escherichia coli* clone in Italy: a whole-genome sequence characterization. Microb Drug Resist.

[9] Liu Y, Feng Y, Wu W, Xie Y, Wang X, Zhang X, Chen X, Zong Z (2015). First report of OXA-181-producing *Escherichia coli* in China and characterization of the isolate using whole-genome sequencing. Antimicrob Agents Chemother.

[10] Schaufler K, Semmler T, Wieler LH, Wöhrmann M, Baddam R, Ahmed N, Müller K, Kola A, Fruth A, Ewers C (2016). Clonal spread and interspecies transmission of clinically relevant ESBL-producing *Escherichia coli* of ST410—another successful pandemic clone?. FEMS Microbiol.

[11] Patiño-Navarrete R, Rosinski-Chupin I, Cabanel N, Gauthier L, Takissian J, Madec J-Y, Hamze M, Bonnin RA, Naas T, Glaser P (2020). Stepwise evolution and convergent recombination underlie the global dissemination of carbapenemase-producing *Escherichia coli*. Genome Med.

[12] Yoon E-J, Yang JW, Kim JO, Lee H, Lee KJ, Jeong SH (2018). Carbapenemase-producing Enterobacteriaceae in South Korea: a report from the national laboratory surveillance system. Future Microbiol.

[13] Kyung SM, Choi S-W, Lim J, Shim S, Kim S, Im YB, Lee N-E, Hwang C-Y, Kim D, Yoo HS (2022). Comparative genomic analysis of plasmids encoding metallo-β-lactamase NDM-5 in Enterobacterales Korean isolates from companion dogs. Sci Rep.

[14] Hong JS, Song W, Park H-M, Oh J-Y, Chae J-C, Han J-I, Jeong SH (2019). First detection of New Delhi metallo-β-lactamase-5-producing *Escherichia coli* from companion animals in Korea. Microb Drug Resist.

[15] D’Costa VM, King CE, Kalan L, Morar M, Sung WW, Schwarz C, Froese D, Zazula G, Calmels F, Debruyne R (2011). Antibiotic resistance is ancient. Nature.

[16] Forsberg KJ, Reyes A, Wang B, Selleck EM, Sommer MO, Dantas G (2012). The shared antibiotic resistome of soil bacteria and human pathogens. Science.

[17] Bengtsson-Palme J, Larsson D (2015). Antibiotic resistance genes in the environment: prioritizing risks. Nat Rev Microbiol.

[18] Finley RL, Collignon P, Larsson DJ, McEwen SA, Li X-Z, Gaze WH, Reid-Smith R, Timinouni M, Graham DW, Topp E (2013). The scourge of antibiotic resistance: the important role of the environment. Clin Infect Dis.

[19] CLSI (2017) M100–S23 Performance Standards for Antimicrobial Susceptibility Testing; Twenty-Third Informational Supplement An informational supplement for global application developed through the Clinical and Laboratory Standards Institute consensus process, 27th edn. Clinical and Laboratory Standards Institute, Wayne

[20] Poirel L, Walsh TR, Cuvillier V, Nordmann P (2011). Multiplex PCR for detection of acquired carbapenemase genes. Diagn Microbiol Infect Dis.

[21] Altschul SF, Gish W, Miller W, Myers EW, Lipman DJ (1990). Basic local alignment search tool. J Mol Biol.

[22] Wirth T, Falush D, Lan R, Colles F, Mensa P, Wieler LH, Karch H, Reeves PR, Maiden MC, Ochman H (2006). Sex and virulence in *Escherichia coli*: an evolutionary perspective. Mol Microbiol.

[23] Zhou Z, Alikhan N-F, Mohamed K, Fan Y, Achtman M, Brown D, Chattaway M, Dallman T, Delahay R, Kornschober C (2020). The EnteroBase user’s guide, with case studies on *Salmonella* transmissions, *Yersinia pestis* phylogeny, and *Escherichia* core genomic diversity. Genome Res.

[24] Wick RR, Judd LM, Holt KE (2019). Performance of neural network basecalling tools for Oxford Nanopore sequencing. Genome Biol.

[25] https://github.com/rrwick/Filtlong.

[26] Kolmogorov M, Yuan J, Lin Y, Pevzner PA (2019). Assembly of long, error-prone reads using repeat graphs. Nat Biotechnol.

[27] Wick RR, Judd LM, Gorrie CL, Holt KE (2017). Unicycler: resolving bacterial genome assemblies from short and long sequencing reads. PLoS Comput Biol.

[28] Hunt M, Silva ND, Otto TD, Parkhill J, Keane JA, Harris SR (2015). Circlator: automated circularization of genome assemblies using long sequencing reads. Genome Biol.

[29] Walker BJ, Abeel T, Shea T, Priest M, Abouelliel A, Sakthikumar S, Cuomo CA, Zeng Q, Wortman J, Young SK (2014). Pilon: an integrated tool for comprehensive microbial variant detection and genome assembly improvement. PLoS ONE.

[30] Simão FA, Waterhouse RM, Ioannidis P, Kriventseva EV, Zdobnov EM (2015). BUSCO: assessing genome assembly and annotation completeness with single-copy orthologs. Bioinformatics.

[31] Seemann T (2014). Prokka: rapid prokaryotic genome annotation. Bioinformatics.

[32] Buchfink B, Reuter K, Drost H-G (2021). Sensitive protein alignments at tree-of-life scale using DIAMOND. Nat Methods.

[33] Conesa A, Götz S, García-Gómez JM, Terol J, Talón M, Robles M (2005). Blast2GO: a universal tool for annotation, visualization and analysis in functional genomics research. Bioinformatics.

[34] Dimmer EC, Huntley RP, Alam-Faruque Y, Sawford T, O’Donovan C, Martin MJ, Bely B, Browne P, Mun Chan W, Eberhardt R (2012). The UniProt-GO annotation database in 2011. Nucleic Acids Res.

[35] O’Leary NA, Wright MW, Brister JR, Ciufo S, Haddad D, McVeigh R, Rajput B, Robbertse B, Smith-White B, Ako-Adjei D (2016). Reference sequence (RefSeq) database at NCBI: current status, taxonomic expansion, and functional annotation. Nucleic Acids Res.

[36] Center for Genomic Epidemiology. http://cge.cbs.dtu.dk/services/all.php. Accessed 21 Aug 2022

[37] Stothard P, Wishart DS (2005). Circular genome visualization and exploration using CGView. Bioinformatics.

[38] Kaas RS, Leekitcharoenphon P, Aarestrup FM, Lund O (2014). Solving the problem of comparing whole bacterial genomes across different sequencing platforms. PLoS ONE.

[39] Tamura K, Stecher G, Kumar S (2021). MEGA11: molecular evolutionary genetics analysis version 11. Mol Biol Evol.

[40] Letunic I, Bork P (2016). Interactive tree of life (iTOL) v3: an online tool for the display and annotation of phylogenetic and other trees. Nucleic Acids Res.

[41] Oelschlaeger T, Zhang D, Schubert S, Carniel E, Rabsch W, Karch H, Hacker J (2003). The high-pathogenicity island is absent in human pathogens of *Salmonella enterica* subspecies I but present in isolates of subspecies III and VI. J Bacteriol.

[42] Feng Y, Liu L, Lin J, Ma K, Long H, Wei L, Xie Y, McNally A, Zong Z (2019). Key evolutionary events in the emergence of a globally disseminated, carbapenem resistant clone in the *Escherichia coli* ST410 lineage. Commun Biol.

[43] Smith JL, Fratamico PM, Gunther NW (2007). Extraintestinal pathogenic *Escherichia coli*. Foodborne Pathog Dis.

[44] Russo TA, Johnson JR (2000). Proposal for a new inclusive designation for extraintestinal pathogenic isolates of *Escherichia coli*: ExPEC. J Infect Dis.

[45] Knothe H, Shah P, Krcmery V, Antal M, Mitsuhashi S (1983). Transferable resistance to cefotaxime, cefoxitin, cefamandole and cefuroxime in clinical isolates of *Klebsiella pneumoniae* and *Serratia marcescens*. Infection.

[46] Pitout JD (2010). Infections with extended-spectrum β-lactamase-producing Enterobacteriaceae. Drugs.

[47] Pitout JD (2012). Extraintestinal pathogenic *Escherichia coli*: a combination of virulence with antibiotic resistance. Front Microbiol.

[48] Cummins EA, Snaith AE, McNally A, Hall RJ (2021). The role of potentiating mutations in the evolution of pandemic *Escherichia coli* clones. Eur J Clin Microbiol Infect Dis.

[49] Gauthier L, Dortet L, Cotellon G, Creton E, Cuzon G, Ponties V, Bonnin RA, Naas T (2018). Diversity of carbapenemase-producing *Escherichia coli* isolates in France in 2012–2013. Antimicrob Agents Chemother.

[50] Schubert S, Picard B, Gouriou S, Heesemann J, Denamur E (2002). *Yersinia* high-pathogenicity island contributes to virulence in *Escherichia coli* causing extraintestinal infections. Infect Immun.

[51] Schubert S, Cuenca S, Fischer D, Heesemann J (2000). High-pathogenicity island of *Yersinia pestis* in enterobacteriaceae isolated from blood cultures and urine samples: prevalence and functional expression. J Infect Dis.

