# Supplementary material for: Genomic molecular epidemiology of carbapenemase-producing Escherichia coli ST410 isolates by complete genome analysis
Source: Vet Res. 2023 Sep 1;54:72. doi: 10.1186/s13567-023-01205-6 (PMC10472685; doi:10.1186/s13567-023-01205-6)
Supplement: Supplementary file 1 — Additional file 1: MIC profiles of the ST410 strains. [file 13567_2023_1205_MOESM1_ESM.docx]

**Additional file 1. MIC profiles of the ST410 strains.**

| **Antibiotics** | **MIC (μg/mL)** | | | |
| --- | --- | --- | --- | --- |
|  | **DMCPEC2** | **DMCPEC3** | **DMCPEC7** | **NB7CPEC** |
| Ertapenem | 64 | 32 | 64 | 128 |
| Imipenem | >256 | >256 | >256 | 16 |
| Meropenem | 64 | 32 | 32 | 64 |
| Ampicillin | >256 | >256 | >256 | >256 |
| Ceftazidime | >256 | >256 | >256 | >256 |
| Cefepime | >256 | >256 | >256 | >256 |
| Chloramphenicol | 16 | 64 | 16 | 8 |
| Colistin | 0.19 | 0.5 | 0.19 | ≤0.25 |
| Gentamicin | 96 | 96 | 48 | >256 |
| Tobramycin | 6 | 4 | 4 | >256 |
| Tetracycline | >256 | >256 | >256 | >256 |
| Trimethoprim | 0.5 | >256 | 0.5 | >256 |
