# Supplementary material for: Genomic molecular epidemiology of carbapenemase-producing Escherichia coli ST410 isolates by complete genome analysis
Source: Vet Res. 2023 Sep 1;54:72. doi: 10.1186/s13567-023-01205-6 (PMC10472685; doi:10.1186/s13567-023-01205-6)
Supplement: Supplementary file 2 — Additional file 2: Whole-genome profiles of sequenced E. coli strains. [file 13567_2023_1205_MOESM2_ESM.docx]

**Additional file 2. The whole genome profiles of sequenced *E. coli* strains.**

| **Strain** | **Contigs** | **Contig max length** | **N50** | **Contig total length** | **Total reads** | **Mapped reads** | **Mapping rate (%)** |
| --- | --- | --- | --- | --- | --- | --- | --- |
| **DMCPEC2** | 3 | 4 832 084 | 4 832 084 | 4 955 448 | 25 559 802 | 25 057 299 | 98.03 |
| **DMCPEC3** | 3 | 4 736 397 | 4 736 397 | 4 865 378 | 28 353 967 | 27 714 214 | 97.74 |
| **DMCPEC7** | 3 | 4 715 539 | 4 715 539 | 4 838 903 | 26 273 875 | 25 071 369 | 95.42 |
| **NB7CPEC** | 5 | 4 759 653 | 4 759 653 | 5 056 577 | 29 304 526 | 28 919 763 | 98.69 |

The whole genome records of sequenced *E. coli* strains. The complete genome was assembled and identified conducting long-read Nanopore MinION sequence and supplemented with short-read Illumina NovaSeq 6000 reads. Alignment summary and assembly results was displayed to verify high quality of assembled whole genome sequence.
