# Supplementary material for: Genomic molecular epidemiology of carbapenemase-producing Escherichia coli ST410 isolates by complete genome analysis
Source: Vet Res. 2023 Sep 1;54:72. doi: 10.1186/s13567-023-01205-6 (PMC10472685; doi:10.1186/s13567-023-01205-6)
Supplement: Supplementary file 5 — Additional file 5: Quality of SNP extraction analysis, including input parameters and identity of each strain with the reference. [file 13567_2023_1205_MOESM5_ESM.docx]

**Additional file 5. The quality of SNP extraction analysis, including input parameters and identity of each strains with the reference.**

**Input Parameters**

**Minimum depth at SNP positions:** 10

**Relative depth at SNP positions:** 10

**Minimum distance between SNPs (prune):** 10

**Minimum SNP quality:** 30

**Minimum read mapping quality:** 25

**Minimum Z-score:** 1.96

Percentage of reference genome covered by all isolates: 88.0893208410978

4 284 225 positions were found in all analyzed genomes.

Size of reference genome: 4 863 501


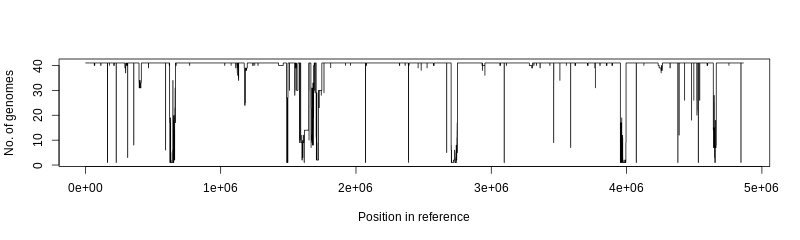


| **Strain** | **Valid positions** | **Identity with reference (%)** |
| --- | --- | --- |
| WCHEC035148 | 4 576 879 | 94.1066733614324 |
| DMCPEC7 | 4 561 611 | 93.7927431288695 |
| UKDogLiverpool | 4 631 169 | 95.2229474199759 |
| ST410 | 4 593 163 | 94.4414938950357 |
| DMCPEC3 | 4 569 415 | 93.9532036695376 |
| FDAARGOS_433 | 4 565 681 | 93.8764277009504 |
| YD786 (Ref) | 4 863 501 | 100 |
| AMA1167 | 4 615 276 | 94.8961663624619 |
| 1EC187 | 4 636 939 | 95.3415862359235 |
| DC71 | 4 602 718 | 94.6379573068865 |
| AR437 | 4 554 893 | 93.6546121816362 |
| SCEC020001 | 4 695 146 | 96.5383989845998 |
| BA22372 | 4 558 059 | 93.719709320508 |
| PT109 | 4 681 358 | 96.2548995055208 |
| 4238 | 4 650 237 | 95.6150106682408 |
| WCHEC025943 | 4 619 908 | 94.9914063963388 |
| BR12DEC | 4 669 445 | 96.0099525012948 |
| DMCPEC2 | 4 595 451 | 94.4885381950163 |
| EcMAD1 | 4 596 443 | 94.5089350243785 |
| 042 | 4 568 513 | 93.9346573589684 |
| EC-TO75 | 4 694 160 | 96.5181255231571 |
| 124 | 4 584 427 | 94.2618702042006 |
| RUT3575 | 4 625 130 | 95.0987776089693 |
| WCHEC020032 | 4 548 708 | 93.5274404179212 |
| AR434 | 4 610 245 | 94.7927223619364 |
| 38 | 4 609 699 | 94.7814958812592 |
| AR24.2b | 4 542 863 | 93.4072595029794 |
| RL465 | 4 630 808 | 95.2155247834842 |
| A1-181 | 4 592 386 | 94.4255177494566 |
| MB98 | 4 726 858 | 97.1904395619534 |
| K71-77 | 4 671 973 | 96.0619315180566 |
| A1-180 | 4 591 429 | 94.4058405662916 |
| SCEC020026 | 4 628 215 | 95.1622092809275 |
| EC25 | 4 674 785 | 96.1197499496762 |
| Ecol_517 | 4 694 144 | 96.5177965420383 |
| KBN10P04869 | 4 615 276 | 94.8961663624619 |
| NB7CPEC | 4 600 647 | 94.5953748133289 |
| Es_ST410_NW1_NDM_09_2017 | 4 623 544 | 95.0661673555737 |
| WCHEC020031 | 4 610 969 | 94.8076087575596 |
| IVRIKolCP4 | 4 562 974 | 93.8207682079226 |
| P24_WM1_05.20 | 4 643 517 | 95.4768385983677 |
